# Information generation as a functional basis of consciousness

**DOI:** 10.1093/nc/niz016

**Published:** 2019-11-29

**Authors:** Ryota Kanai, Acer Chang, Yen Yu, Ildefons Magrans de Abril, Martin Biehl, Nicholas Guttenberg

**Affiliations:** Basic Research Group, Araya, Inc., P.O. Box 577 ARK Mori Building 24 F, 1-12-32 Akasaka, Minato-ku, Tokyo, 107-6024, Japan

**Keywords:** consciousness, imagery, qualia, theories and models, computational modeling

## Abstract

What is the biological advantage of having consciousness? Functions of consciousness have been elusive due to the subjective nature of consciousness and ample empirical evidence showing the presence of many nonconscious cognitive performances in the human brain. Drawing upon empirical literature, here, we propose that a core function of consciousness be the ability to internally generate representations of events possibly detached from the current sensory input. Such representations are constructed by generative models learned through sensory-motor interactions with the environment. We argue that the ability to generate information underlies a variety of cognitive functions associated with consciousness such as intention, imagination, planning, short-term memory, attention, curiosity, and creativity, all of which contribute to non-reflexive behavior. According to this view, consciousness emerged in evolution when organisms gained the ability to perform internal simulations using internal models, which endowed them with flexible intelligent behavior. To illustrate the notion of information generation, we take variational autoencoders (VAEs) as an analogy and show that information generation corresponds the decoding (or decompression) part of VAEs. In biological brains, we propose that information generation corresponds to top-down predictions in the predictive coding framework. This is compatible with empirical observations that recurrent feedback activations are linked with consciousness whereas feedforward processing alone seems to occur without evoking conscious experience. Taken together, the information generation hypothesis captures many aspects of existing ideas about potential functions of consciousness and provides new perspectives on the functional roles of consciousness.

**Highlights**

- Drawing upon empirical research into consciousness, we propose a hypothesis that a function of consciousness is to internally generate counterfactual representations detached from the current sensory events.
- Interactions with generated representations allow an agent to perform a variety of non-reflexive behaviors associated with consciousness such as cognitive functions enabled by consciousness such as intention, imagination, planning, short-term memory, attention, curiosity, and creativity.
- Applying the predictive coding framework, we propose that information generation is performed by top-down predictions in the brain.
- The hypothesis suggests that consciousness emerged in evolution when organisms gained the ability to perform internal simulations using generative models.

## What Is the Function of Consciousness?

The notion that consciousness is a natural phenomenon suggests that consciousness is subject to a set of universal laws of nature, and a scientific theory of consciousness should correctly identify physical or information-theoretic conditions in which consciousness occurs (Chalmers 1993). This implies that biological brains are not the only medium that gives rise to consciousness. Any physical system that satisfies the necessary and sufficient condition for generating consciousness should possess internal experience (Searle 2017). However, those conditions have been difficult to determine due to the subjective nature of conscious experience.

Consciousness has both functional and subjective aspects (Block 1995). The functional aspect, also known as access consciousness, is the objectively observable aspect of consciousness, which are amenable to scientific scrutiny, while the subjective aspect, known as phenomenal consciousness, is not directly observable except for the person experiencing that conscious state. Understanding how phenomenal consciousness occurs within physical systems is known as the Hard problem of consciousness (Chalmers 1997) and is considered to be one of the toughest problems in science. Much of the interest devoted to possible solutions to the Hard problem often results in the views that consciousness is epiphenomenal, i.e., that subjective experiences exist only as a by-product of information processing without playing any functional role (Velmans 1991). Instead of directly addressing the Hard problem, a possibly more productive direction might be to consider putative functions of consciousness, namely, cognitive functions that require consciousness in the sense of being awake and able to report stimulus contents with confidence. Here, we consider consciousness both in terms of the state of consciousness (e.g. wakefulness) and the contents of consciousness (e.g. awareness of specific sensory stimuli).

In this Opinion article, we examine possible functions of consciousness in the current literature of neuroscience and psychology, and propose that information generation of possibly counterfactual representations is the core faculty of consciousness. Traditionally, many high-level cognitive functions have been associated with consciousness. However, more recent studies have uncovered cognitive functions such as attention (Kanai *et al.* 2006; Bahrami *et al.* 2007; Koch and Tsuchiya 2007; Hsieh *et al.* 2011), working memory (Soto *et al.* 2011; King *et al.* 2016), and executive control (Lau and Passingham 2007) can be performed in the absence of conscious awareness (Lin and He 2009). Those empirical findings have made it difficult to pin down cognitive functions that require consciousness. What then is the critical function that requires consciousness? In the literature, there have been a few suggestions for cognitive functions that seem to require conscious awareness (Dehaene *et al.* 2017).

## Consciousness Is for Non-Reflexive Behavior

### Reflexive versus non-reflexive behavior as a marker of consciousness

In clinical settings, the ability to execute non-reflexive behavior is taken as a sign of the presence of consciousness when distinguishing disorders of consciousness (Piccinini and Craver 2011): the minimally conscious state (MCS) in which the patient is awake and retains awareness of the self and environment, and the unresponsive wakefulness syndrome (Laureys *et al.* 2010) in which the patient is awake but lacks awareness of the self and environment. According to a modern guideline, MCS is characterized by the presence of cognitively mediated behavior, which is differentiated from reflexive behavior (Giacino *et al.* 2002). That is, in disorders of consciousness, the presence of consciousness depends on intentional, deliberate behavior as opposed to automated, reflexive behavior triggered solely by the properties of the current sensory input. While identification of non-reflexive behavior is difficult if we were to rely only on behavioral characteristics displayed by a patient, this clinical definition appeals to our intuition that consciousness is needed in non-reflexive behavior.

### Bridging a temporal gap in classical conditioning

Trace conditioning is a kind of classical conditioning which pairs conditioned stimulus (CS) such as a tone with unconditioned stimulus (US), which evoked a response such as an eyeblink from air puffs to the eye. Classical conditioning is said to be a trace conditioning when CS and US do not overlap in time as opposed to a delay conditioning in which CS and US overlap in time. Crucially, empirical evidence suggests that successful trace conditioning requires awareness of the relationship between CS and US, whereas delay conditioning occurs automatically regardless of whether the subject became aware of the relationship (Clark and Squire 1998; Clark *et al.* 2002).

The important difference is that in trace conditioning, the subject needs to bridge the temporal gap between CS and US by retaining the information of CS over a blank period. However, the brain cannot prepare automated circuits to associate all possible arbitrary combinations of temporally segregated events, because the number of combinations increases when we consider many different gap durations. Specifically, the information about the CS needs to be maintained over time in the brain to be associated with the neural activity evoked by the US. Therefore, the CS needs to be selected as such from many other possible stimuli. That is to say, the subject needs to form a hypothesis about the relationship between CS and US. This contrasts with delay conditioning in which the temporal overlaps allow neural activities evoked by CS and US to directly interact with each other without an additional mechanism to sustain information from the past. As such, the subject does not need to pick a stimulus as a CS to maintain over a short time period. These considerations based on findings in associative learning studies suggest that a possible function of consciousness is to bridge a temporal gap by selecting a small number of events.

### Delayed response in a patient with agnosia

Another clue comes from experiments on the agnosia patient DF who had impairments in conscious object recognition (Goodale *et al.* 1991). When she was asked to indicate the orientation of a slanted slit verbally or by adjusting a handle, she could not report the orientation, suggesting that she had no awareness of the orientation. However, she could post a letter through the slit by adjusting the orientation of the letter in the right angle, suggesting that she could use the orientation information for guiding action. This is a classic example that led to the proposal that the ventral pathway (where the patinet DF had a damage) is for conscious vision, whereas the dorsal pathway is for guiding action without necessarily evoking conscious experience.

Crucially, when she was shown the slot first, and then the light was turned off so that she would have to wait for a few seconds before acting, then she failed to reach the slot correctly (Goodale *et al.* 1994; but see Hesse and Schenk 2014). This suggests that the unconscious action system needs to be guided online by visual information and to act on offline information retained from the recent past requires the ability to consciously perceive the shape. In other words, “online systems” that process information real time works without awareness, but to maintain information over time, consciousness is necessary.

These examples suggest that a possible function of consciousness might be to maintain sensory information in short-term memory in a flexible, usable form over a period of time after the stimulus is no longer present. Indeed, based on such observations, Koch proposed a delay test for consciousness in biological systems where the presence of consciousness is assessed by the ability to perform a task when a temporal gap is inserted between a stimulus and an action (Koch 2004).

This is in line with the second and the third laws of the three laws of qualia—a framework proposed by Ramachandran and Hirstein (1997). “Qualia” refers to the qualitative aspect of conscious experience, which is sometimes characterized as the redness of a sunset or the painfulness of headache (Kanai and Tsuchiya 2012). In the three laws of qualia, qualia are characterized by three functional properties, namely, (i) irrevocability, (ii) flexibility, and (iii) short-term memory. Irrevocability refers to the observation that we cannot override the contents of experience at will via top-down attention. Flexibility implies the fact that once sensory events are consciously registered, the contents can be used for multiple, open-ended purposes unlike reflexive behavior. Short-term memory refers to the fact that the content of consciousness remains for a sufficient time to allow interaction with the executive system for making choices.

### Counterfactual predictions for intention and planning

Another important aspect of consciousness pertains to motor behavior such as intention or planning. Intention can be formulated as predictions about the consequences of actions before those actions are executed (Jeannerod 1994). For example, I can predict my sensory inputs from visual and somatosensory systems that would arise if I were to make a particular action such as waving my hand in front of my face even without actually performing the movement. I can also think about going to the kitchen to make coffee without actually standing up from the couch in the living room. These instances of intention or planning are counterfactual predictions in the sense that planned actions have not materialized in the reality while imagining a possible future.

The importance of consciousness in planning has been argued in the literature. More than 20 years ago, Crick and Koch discussed plausible functions of visual awareness are to produce the best interpretation of the current scene, and to make the information available for voluntary actions after a delay (Crick and Koch 1995, 1998). Based on this view, they once proposed that neurons in the primary visual cortex (V1) do not directly produce conscious experience (Crick and Koch 1995). The rationale for the V1 hypothesis comes from the consideration that the biological utility of conscious perception is to make the sensory information available for planning and executing voluntary motor outputs. When we consciously experience sensory information (e.g. seeing a red ball), we can use that information for multiple purposes such as planning future actions. Crucially, the V1 hypothesis predicted that neuronal activities in V1 do not directly contribute to conscious experience because V1 neurons do not directly project to the prefrontal cortex implicated in the function of planning. While the position of one of the original proposers may have changed (Koch *et al.* 2016), we believe that the rationale that a functional consequence of conscious perception is the availability of the information for future planning is still valid independent of his current position.

The idea is also compatible with the current global workspace theory in which the role of consciousness is to make sensory information shared across multiple cortical regions including the fronto-parietal networks (Baars 1997, 2005; Dehaene *et al.* 1998; Dehaene and Naccache 2001). The key idea is that consciousness allows one to utilize the content of conscious perception for flexible purposes (Earl 2014). These hypotheses put emphasis on short-term memory, working memory, planning and executive control as functional capabilities endowed by consciousness. In other words, a function of consciousness is to make information globally available across the system.

Another possible role of consciousness is metacognition. A wide range of cognitive functions are known to be performed without awareness. As psychophysical and neurological studies have shown, objective performance in a visual task can be above chance while the subject claims to have no awareness of seeing a stimulus. Thus, it is thought that a higher-level, meta-representation of a first-order representation of a sensory stimulus should be needed for consciousness. This idea is closely related to the notion of higher-order theories of consciousness (Carruthers 2005; Rosenthal 2006; Lau and Rosenthal 2011). In line with higher-order theories, confidence ratings are often used as a proxy measure for the presence of conscious perception (Fleming and Lau 2014; Sherman *et al.* 2015).

These two aspects of consciousness, i.e., broadcasting of information and metacognition, have been summarized in a recent paper (Dehaene *et al.* 2017) as C1 and C2, respectively. The functions of consciousness discussed in this Opinion article mainly concerns C1, and our information generation hypothesis proposes that what underlies a variety of cognitive functions such as short-term memory, intention, and planning is the capability of generating fictional representations using internal, sensorimotor models.

Taken together, we propose that a key function of consciousness is to allow non-reflexive behavior such as responding after a delay, or executing an action based on internally generated plans. Is there a computational mechanism that is common across a variety of non-reflexive behavior?

## Counterfactual Information Generation Theory of Consciousness

Here, we suggest that a common thread across those examples of non-reflexive behavior is information generation. It’s the ability to internally generate sensory representations that are not direct reflections of the current sensory input. They could be counterfactual representations since information maintained over time in short-term memory from the past or predictions of sensory consequences of unexecuted future actions do not necessarily correspond to events actually happening in the present environment.

Our hypothesis is that this ability to generate possibly counterfactual representations using internal models learned through interactions with the environment is the function of consciousness. This hypothesis, which we call information generation hypothesis, helps us understand functional advantages of consciousness and predict under what kind of tasks consciousness would be required.

The ability to generate internal representations independent of current sensory inputs allows an agent to engage with possibly counterfactual states not happening at the present moment. This enables an agent to detach itself from the environment and perform non-reflexive behavior such as planning future actions through mental simulations of the environment (Grush 2004). Furthermore, it allows an agent even to learn from fictional scenarios it has never experienced (Ha and Schmidhuber 2018). This capability provides an enormous advantage to organisms for survival.

### Counterfactuals in model-based reinforcement learning

The functional advantage of internal simulation is also apparent in reinforcement learning. In the literature of reinforcement learning, learning strategy is divided into model-free and model-based (Dayan and Berridge 2014). Model-free reinforcement learning such as Q-learning learns to derive the best action under the current state but does not involve direct learning of the structure of the environment. Model-based reinforcement learning captures the sequential contingencies of events and actions, namely, the predictive model of future states for a given action. This model-based approach allows an agent to use the knowledge of the structure of the environment to plan a course of action. For example, a bird’s eye model of a maze would allow an agent to plan for the optimal path through an internal simulation. The model-based approach thus affords an agent with the ability to compute an optimal sequence of actions through mental simulation using their internal models. In both biological and artificial intelligence, generative models of action-state sequences play an essential role in model-based reinforcement learning. For example, Dyna proposed by Sutton (1991) adopts the idea that planning is “trying things in your head.” Crucially, the model-based approach allows an agent to adapt to new goals flexibly because it can use the internal model to optimize its behavior without trial and error.

### Intrinsic motivation, saliency, and infotaxis

Predictions of future states are useful for sampling information from the environment efficiently. This is because such generated future states allow an agent to estimate various statistical quantities of interest in a given context. For example, an agent can compute in a model-based manner what would be the expected information gain (Schmidhuber 1991, 2010) or other forms of intrinsic motivation (Oudeyer 2007) in response to future (and therefore counterfactual) actions. The framework of active inference (Friston *et al.* 2012, 2013, 2015) promises to treat future actions in a similar way as other counterfactual events like hidden causes and infer them by conditioning on an optimized utility function. While this utility function usually corresponds to expected free energy it has been shown that the same approach can be equally applied to various other intrinsic motivations (Biehl *et al.* 2018). This suggests that the information generation perspective may be able to naturally incorporate current research on intrinsic motivations as well.

In the context of attention and eye movement, expected information gain can be considered as a saliency map (Friston *et al.* 2012). Such formulation is considered model-based in the sense that the saliency (i.e. expected decrease in the entropy of hidden states) is computed based on a generative model of the sensory input given the underlying hidden cause. Infotaxis is another such example where search behavior is guided by expected information gain given an action and is likely to be found in many creatures (Vergassola *et al.* 2007). These model-based search strategies are important when signals are sparse, and highlight the utility of counterfactual predictions in biological systems (Friston *et al.* 2015).

Taken together, our analysis suggests that the ability to generate information enables an agent to perform mental simulations for planning future action sequences, which would be otherwise difficult only with a collection of reflexive behaviors. This ability underlies model-based reinforcement learning and intrinsic motivations such as curiosity and infotaxis.

## What Does It Mean to Internally Generate Information?

So far, we have argued that the ability to generate counterfactual representations is the core function of consciousness, allowing an agent to perform non-reflexive behaviors. However, we have not specified what kind of neural processing should be considered as generating information.

Here, we argue that information generation can be seen as production of sensory representations using internal models. This is achieved by reversing the process of representation learning, which is projection from sensory inputs to internal models. In what follows, we will illustrate the concept of information generation using modern approaches to constructing generative models with deep neural networks.

### Generative models in deep neural networks

Many sophisticated methods such as generative adversarial networks (Goodfellow *et al.* 2014; Gulrajani *et al.* 2017; Hindupur 2017) or variational autoencoders (VAEs) (Kingma and Welling 2013) for training models for generating sensory representations have been developed in recent years, but they have not received sufficient attention in the context of neuroscience. While the concept of learning a generative model—statistical model of the process of how sensory data are generated from a set of hidden causes—is common in interpreting computational principles of the brain, what it means for the brain to use generative models for producing representations has rarely been considered. Here, we illustrate the idea of information generation using the (variational) autoencoder as a metaphor and relate it to the predictive coding architecture in the brain.

**Figure 1.**
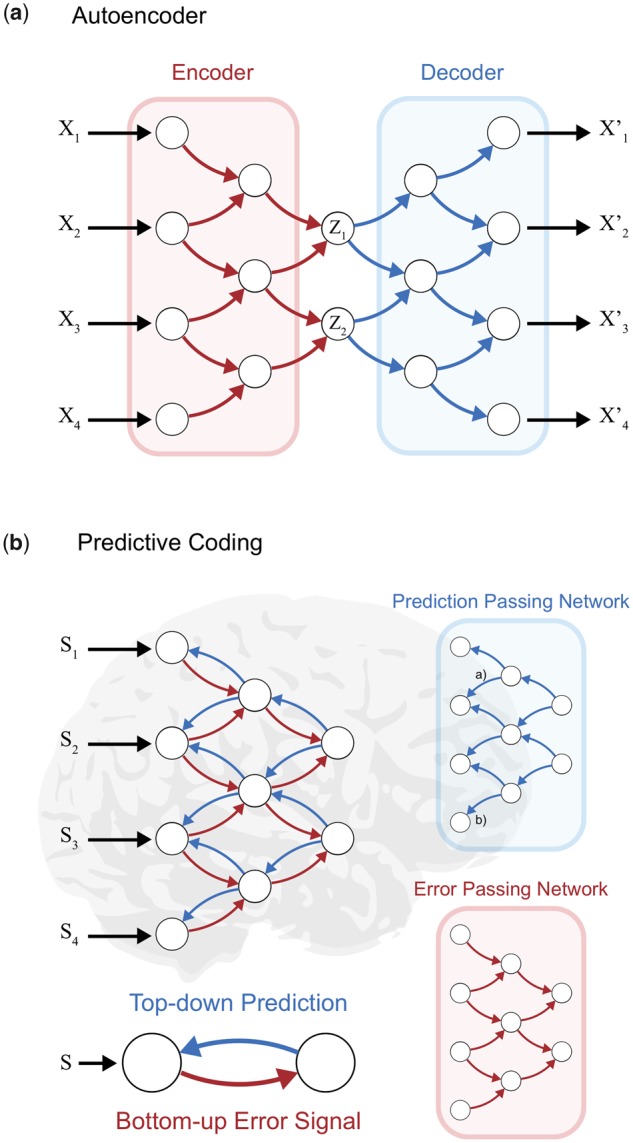
Comparison of information generation in (**a**) autoencoder and (**b**) predictive coding. (a) In autoencoders, the encoder part (shown in red) compresses sensory information to compact representations in a latent space. This representation is decoded into sensory data format. The decoder (shown in blue) can be used for counterfactual information generation using a seed chosen from the latent space. The variables *z*_1_ and *z*_2_ represent the latent variables. (b) In the predictive coding hypothesis of the biological brain, bottom-up error signals (shown in red) correspond to data compression or encoding in autoencoders, whereas top-down predictions (shown in blue) correspond to information generation. Note that in predictive coding, our hypothesis predicts that the top-down predictions generate conscious experience

VAEs are a class of neural networks consisting of an encoder/inference network and a decoder/generative network (Fig. 1a). The encoder works as data compression by transforming the input data (e.g. image) to abstract, latent representations of low dimensionality (i.e. parameters of a Gaussian distribution). The decoder then converts the abstract representation back to the original data space as the output. The networks are trained so that the output matches the input as closely as possible, namely, maximizing the lower bound of the probability of generating real data samples. Once trained successfully, the decoder network can be used independently of the encoder network, and thus without the input, to generate representations in the data space (e.g. see Fig. 1). This property of the decoder network enables production of novel and counterfactual representations based on previous experiences. The loss function of VAE is known as the evidence lower bound and is related to in Friston’s free energy principle (Friston 2009, 2010).

Under the computational scheme of VAEs, the decoder/generative and encoder/inference networks correspond to the generation and processing of information, respectively (Fig. 1a). The decoder/generative network can be used for generating representations in the data space by choosing a state in the latent space, which allows production of counterfactual representations including those the agent has never encountered before. From the architecture of VAEs, we can formulate information generation in terms of mapping from an abstract low-dimensional representation to a high-dimensional representation in the data (i.e. sensory) space.

### Predictive coding framework of the brain

However, the architecture of the VAEs does not resemble the networks found in the biological brain. How do those encoder and decoder networks in VAEs correspond to the network architectures in biological brains? To establish correspondences with the brain, we consider hierarchical predictive coding (Rao and Ballard 1999; Friston and Kiebel 2009; Hohwy 2014). According to this view, representations at a higher layer serve as a prediction for a lower layer, and this is communicated via top-down signals, whereas prediction errors (i.e. the discrepancy between the top-down prediction and bottom-up signals) are communicated back to the higher layer to update the prediction.

At a conceptual level, the reciprocal interactions between top-down prediction and bottom-up prediction error in the predictive coding framework can be roughly mapped to the decoder and encoder in VAEs, respectively (Fig. 1b). While whether the brain utilizes the predictive coding architecture is still a matter of debate, and there are multiple ways to implement similar architectures (e.g. Heeger 2017), the framework provides a link between the encoder/decoder scheme presented above and possible counterparts in the biological brain. That is, information generation, which we claim to be crucial for consciousness, is mediated by top-down prediction, whereas the bottom-up message passing of prediction errors corresponds to encoding (or data compression). By interpreting feedback predictions as information generation, our current hypothesis predicts that consciousness is generated by the feedback predictions. This is in line with multiple observations that reports of conscious perception is associated with additional feedback activations from higher sensory areas to lower sensory areas (Lamme *et al.* 2000; Supèr *et al.* 2001; Manita *et al.* 2015).

Note, however, that information generation could in principle occur both in feedforward and feedback networks, as the example of VAE consists of purely feedforward networks. Recurrent connections are just a specific case of implementing information generation in the brain and are not a prerequisite for information generation. Therefore, the information generation hypothesis predicts that consciousness could be also produced in simple networks like the VAE as long as they generate information (see next section for a related issue). While we acknowledge that some readers might find it unlikely that such a simple network possesses consciousness, we leave this possibility open for future discussion.

### Information generation from an intrinsic perspective

We have attributed information generation to the decoder/generative network in VAEs and top-down feedback in predictive coding networks. However, these attributions are made from an extrinsic viewpoint. If we are given an arbitrary network of neurons, it would be difficult to determine which part of the network corresponds to a decoder or an encoder. Is there a principled way to determine whether a system is generating information? One noticeable characteristic that distinguishes the decoder network from the encoder network is that in the decoder, the network has a divergent structure from a low-dimensional representation to a higher dimensional representation, whereas in the encoder network, the network has a convergent structure. Further work is needed to determine which part of a network should be considered as generating information from an intrinsic perspective without requiring an extrinsic interpreter.

## Related Theories

In this Opinion paper, we argued that a potential function of consciousness is to generate potentially counterfactual representations, and this function allows flexible response mechanisms for various forms of non-reflexive, goal-directed behavior. As discussed earlier, our idea is compatible with putative functions of consciousness proposed by various authors in the context of the global workspace theory (Dehaene *et al.* 1998; Baars 2005), V1 hypothesis (Crick and Koch 1995), and the three laws of qualia (Ramachandran and Hirstein 1997), in which flexible use of sensory information is thought to be a key element of consciousness. Also, a very similar idea has been proposed by Pennartz that consciousness is for deliberate decision making on goal-directed behavior requiring internal generation of representations (Pennartz 2018). In what follows, we briefly overview other theories that are relevant to our current hypothesis.

### Semantic pointer competition

A notion similar to the decoder networks in our hypothesis has been proposed in the context of the semantic pointer competition (SPC) hypothesis (Thagard and Stewart 2014). SPC proposes that consciousness is supported by representations, semantic pointers, and competition among semantic pointers. In brief, SPC posits that neural representations constructed for each modality (e.g. sensory, motor, and emotional) are combined to form the so-called semantic pointer, which works as a symbol that bundles associated representations coming from multiple modalities. The content of consciousness is determined through competitive mechanisms among semantic pointers towards a limited capacity. In this hypothesis, semantic pointers play two functions. One is to make predictions/inferences about other semantic pointers and the other is to unpack specific contents in the representation coming from a specific modality. A key theoretical difference is that the information generation hypothesis claims that this unpacking process corresponds to the generation of conscious experience, whereas SPC takes the whole architecture consisting of the three components (i.e. representations, semantic pointers, and competition) to be the foundation of consciousness.

### Regret

Frith and Metzinger (2016) proposed that a function of consciousness is associated with the notion of regret. Regret implies extension of the self across time because regret is about the action taken in the past or anticipation of regret in the future. This hypothesis built around the notion of regret also includes other functions of consciousness such as the ability to communicate subjective experience with others, the ability to distinguish the real present from imaginary possible desirable states as used in future planning, and persistence of the self across time through the model of the self. Information generation discussed in our paper should be considered as a way to implement mechanisms to compute regret through production of and interactions with counterfactual past and future. The ability to evaluate counterfactual regret would serve the purpose of learning causal relationship between action and consequences (Pearl and Mackenzie 2018).

### Qualia: factual versus counterfactual representations

Several authors have argued that one important function of conscious experience is the ability to distinguish between representations of factual reality of the here and now and counterfactual possibilities (Gregory 1998; Kanai and Tsuchiya 2012; Frith and Metzinger 2016). While we have emphasized the link between potentially counterfactual representations and consciousness, conscious experience is more vivid for the actual sensory input. How can the information generation hypothesis explain the stronger conscious experience for factual events than those for counterfactual possibilities?

Our interpretation is that conscious experience of the current sensory input is also a product of the internal, generative models. But instead of generating counterfactual events, the same generative networks produce a representation of the current input. The differences in vividness of experience come from the differences in the degree of details produced by the generative models (D’Argembeau and Van der Linden 2006; Dijkstra *et al.* 2017; Pearson 2019). When counterfactual representations are generated (e.g. imagining a face), generative networks can specify only up to a certain level of details, whereas when bottom-up signals are available (e.g. seeing a face), generative networks can utilize those signals to produce representations with finer details. We speculate that this difference in the level of details in internally generated images corresponds to the differences in the vividness between actual and counterfactual experiences and serves the purpose of distinguishing factual and counterfactual representations.

Furthermore, there has been a suggestion that counterfactual representations implicit in the internal models of sensorimotor contingencies contribute to the “presence” of an object of perception (Seth 2014). According to this theory, the perceived presence associated with a stimulus is supported by counterfactually rich generative models that have learned sensorimotor contingencies. Given the importance of perceptual presence in conscious experience of here and now, numerous possibilities stored in the structure of the generative models may shape the ineffable richness of qualia.

However, vividness of experience does not always guarantee that the sensation reflects the reality. In situations such as visual illusions, hallucinations, and dreams, we can have vivid experience of counterfactual events in the sense that they are not veridical to the external physical reality. For example, experiences generated by hallucinations are vivid and therefore should be considered as qualia even though we know that they are internally generated and hence counterfactual. Thus, whether an experience counts as a quale is not determined by whether it is counterfactual or factual, but to what extent details of the experience are produced by generative models. This leads to an interpretation of why dreams feel more vivid than imagination. While dreaming, bottom-up signals would be missing due to the absence of input stimuli. Therefore, top-down generative signals can produce more details without interruptions from bottom-up signals which would be present during awake states. This would lead to higher degrees of details in generated images in dreams and hence more vivid sensation despite the fact that all the images are internally generated.

### Relation to Integrated Information Theory

How is our hypothesis related to Integrated Information Theory (IIT) proposed by Tononi and colleagues (Tononi 2004, 2008; Oizumi *et al.* 2014, 2016; Tononi *et al.* 2016; Tajima and Kanai 2017)? The idea that information generation lies at the core of consciousness is compatible with the main tenet in IIT that consciousness is information. However, the notion of information generation is different between these two theories. In our information generation hypothesis, information generation refers to the offline use of generative models so that the agent can interact with potentially counterfactual situations. Therefore, the hypothesis assumes the presence of an internal model. On the other hand, information generation in the context of IIT is a more general concept in which information is generated due to a specific state of the system which is causally linked with the past and the future and hence possesses information about them. As such, IIT does not assume the presence of an internal model or any specific structure. However, a possible link between the two theories is that a system with the ability to engage in an internal simulation using internal models may also integrate information because internal simulations require a loop structure where generative models are used recursively, and internal models need to reflect the complex dynamics of the environment. Further research is needed to explore this possibility to establish the relationship between the two theories.

### Algorithmic information theory of consciousness

Another information-theoretic approach to consciousness is Kolmogorov Theory (KT) of consciousness (Ruffini 2017). KT proposes consciousness is generated by compressive models of the world. This is similar to our theory in the sense that specific information processes in the internal models are associated with production of consciousness. However, a key difference from our hypothesis is that KT emphasizes the compression process performed by internal models. However, we argue that the compressive processes are not associated with consciousness, and instead decompression (i.e. generation) is linked with consciousness.

### Origins of conscious mind

While consciousness and intelligence are generally distinguished as separate entities, our hypothesis suggests a possible link between the intelligent behavior endowed by flexible use of internal sensorimotor models and consciousness.

What is the functional benefit of having the ability to generate counterfactual representations? Dennett’s formulation of stages of evolution of minds (Dennett 1996) illustrates the benefit of counterfactuals in evolution and intelligence. Dennett proposed useful distinctions among different stages of the evolution of creatures. These include the following four types of creatures.

The first stage of creatures is called Darwinian. Darwinian creatures are the most primitive form of creatures. The organisms at this stage do not learn from experiences but have a fixed set of behaviors determined by their genetic composition. They adapt to the environment only as a species through natural selection and mutation to their genes. Thus, learning occurs at a population level but not as individual agents.

The second stage is called Skinnerian. Skinnerian creatures—named after the psychologist Skinner—learn from their own experience through associative learning. If a particular action led to a reward (e.g. food), then that action is reinforced, whereas an action that led to an aversive result will be avoided. Skinnerian creatures can adapt to the environment during the lifetime of individual organisms through trial and error. However, there is a limitation in that they can only learn from their own experiences. They would have to learn danger from surviving dangerous situations.

Then, there comes the third stage of creatures—Popperian creatures named after the philosopher Popper. They have internal simulation of the environment and select an action based on predictions about simulated consequences of repertoires of future action sequences. This ability allows Popperian creatures to learn from their simulated, counterfactual experience, and to select reasonable actions even in a new environment when their internal model approximates it reasonably well. Moreover, the ability to form counterfactual representations of sensory consequences contingent upon their future action is the core function of intention. The information generation theory of consciousness suggests that consciousness appears at this stage of the evolution of the mind. In other words, our theory suggests the origin of consciousness is the functional advantage of having counterfactual predictions using an internal model of the environment, as it allows Popperian creatures to simulate consequences of possible actions and avoid executing actions that could be dangerous for survival.

Finally, the fourth stage is called Gregorian. Gregorian creatures can learn from their cultural environment through words and language. This requires the ability to run a simulation of the environment using information they learned through books and the Internet. In Popperian creatures, internal models had to be built upon their own experiences. Gregorian creatures on the other hand can improve their internal models through communication with other agents, which endows them with an additional leverage of intelligence. Here, the ability to internally represent counterfactual states is a prerequisite.

Our current hypothesis predicts that the presence of consciousness should be measured by the presence of internal generative models of the environment and the self. Moreover, the hypothesis predicts that model-based action selection should require consciousness. Is model-based reinforcement learning or other forms of model-based decision making possible without consciousness? One important future direction suggested by these predictions is to establish a formal computational and experimental framework to determine whether a given system relies on information generated by its internal generative models. In the example of VAE, we argued that dimensional expansion might be the essence of information generation. However, we need to define information generation more formally as an intrinsic property of a system. Further work is needed to establish such formalisms.

## Recapitulation

To recapitulate, we set off to identify functions of consciousness by examining cognitive tasks that appear to require consciousness. Based on a few examples such as trace conditioning and delayed, non-reflexive responses, we aimed to extract functional essences underlying those tasks, and hypothesized that a function of consciousness is to bridge the temporal gap by internally generating representations of stimuli that are no longer present in the proximal environment. While the hypothesis emphasized the counterfactual aspect of generated information, it considers the conscious perception of current stimuli also as a product of internal generation. To illustrate the processes underlying information generation, we discussed information generation in the decoder networks in VAEs and speculated that in the brain, feedback connections should correspond to such processes. Finally, we reversed the logic of necessity and sufficiency and postulated a stronger version of our hypothesis that a system with the ability to generate representations detached from the current sensory input must have consciousness.

Note that there are two levels of hypotheses within our information generation hypothesis. A weak version of the hypothesis simply claims that a function associated with conscious experience is to generate and maintain representations of events detached from the current sensory input. This version predicts that tasks in which correct responses are not immediately available thereby requiring interactions with internally generated representations depend on consciousness. In other words, the weaker version claims that information generation is necessary for a system to perform consciousness dependent tasks. As such, the ability to perform a task that require internal generation of information is considered as an indicator of consciousness.

On the other hand, a stronger version of the hypothesis goes further and predicts that machines with similar functionalities would also possess consciousness even if they are relatively simple to build. This implies that all tasks that require counterfactual information generation also require consciousness. Therefore, consciousness and counterfactual information generation cannot be distinguished and we consider them identical. In other words, the stronger version claims that counterfactual information generation is sufficient for a system to be conscious. While readers may find this possibility unlikely, we argue that this possibility should be left open for further studies and the conceptual distinction of the two versions of the hypothesis would be useful for further examining this hypothesis in light of future research.

In the present paper, we discussed functions of consciousness. However, the term “function” was used in two different senses, i.e., teleological functions and functions in the sense of (mechanistic) functionalism. To avoid confusion, we would like to clarify how these two meanings of function are related to the weaker and the stronger claim of the hypothesis discussed above. The weaker claim, namely, the necessity of information generation concerns the teleological, purpose-related functions of consciousness. We discussed task conditions in which the presence of consciousness is required for successful performance and argued that what is common among those conditions is the function to generate potentially counterfactual events internally using generative models. The stronger claim on the other hand concerns a form of functionalism (i.e. mechanistic functionalism) in which specific functional mechanisms of cognition correspond to conscious mind (Piccinini and Craver 2011). Specifically, we proposed that mechanisms of information generation are sufficient for consciousness. While it is premature to provide a formal, mathematical definition of information generation, we expect that there be a specific network structure (i.e. a mechanism) that allows an agent to use generative models recursively in an offline manner.

## Concluding Remarks

In summary, we proposed that the ability to generate possibly counterfactual information underlies a variety of cognitive functions enabled by consciousness such as intention, imagination, planning, short-term memory, attention, curiosity, and creativity, all of which contribute to non-reflexive, behavioral flexibility. We further hypothesized that information generation corresponds to feedback predictions in the brain using the predictive coding framework as a model of the brain. Further studies are needed to formally define information generation as an intrinsic property of biological and artificial systems.

## References

[niz016-B1] Baars BJ. Global workspace theory of consciousness: toward a cognitive neuroscience of human experience. Prog Brain Res 2005;150:45–53. 16186014 10.1016/S0079-6123(05)50004-9

[niz016-B2] Baars BJ. In the theatre of consciousness. Global Workspace Theory, a rigorous scientific theory of consciousness. J Conscious Stud 1997;4:292–309.

[niz016-B3] Bahrami B, Lavie N, Rees G. Attentional load modulates responses of human primary visual cortex to invisible stimuli. Curr Biol 2007;17:509–13. 17346967 10.1016/j.cub.2007.01.070 PMC1885953

[niz016-B4] Biehl M, Guckelsberger C, Salge C, et al Expanding the active inference landscape: more intrinsic motivations in the perception-action loop. Front Neurorobot 2018;12. 10.3389/fnbot.2018.00045 PMC6125413 30214404

[niz016-B5] Block N. On a confusion about a function of consciousness. Behav Brain Sci 1995;18:227–47.

[niz016-B6] Calhoun AJ, Chalasani SH, Sharpee TO Maximally informative foraging by *Caenorhabditis elegans*. Elife 2014;3:e04220. 10.7554/eLife.04220 PMC4358340 25490069

[niz016-B7] Carruthers P. Consciousness: Essays from a Higher-Order Perspective. Oxford, NY: Oxford University Press, 2005.

[niz016-B8] Chalmers DJ. Toward a Theory of Consciousness. Indiana University, 1993.

[niz016-B9] Chalmers DJ. The Conscious Mind: In Search of a Fundamental Theory. New York: Oxford University Press, 1997.

[niz016-B10] Clark RE, Manns JR, Squire LR. Classical conditioning, awareness, and brain systems. Trends Cogn Sci 2002;6:524–31. 12475713 10.1016/s1364-6613(02)02041-7

[niz016-B11] Clark RE, Squire LR. Classical conditioning and brain systems: the role of awareness. Science 1998;280:77–81. 9525860 10.1126/science.280.5360.77

[niz016-B12] Crick F, Koch C. Consciousness and neuroscience. Cerebral Cortex 1998;8:97–107. 9542889 10.1093/cercor/8.2.97

[niz016-B13] Crick F, Koch C. Are we aware of neural activity in primary visual cortex? Nature 1995;375:121–3. 7753166 10.1038/375121a0

[niz016-B14] D’Argembeau A, Van der Linden M. Individual differences in the phenomenology of mental time travel: the effect of vivid visual imagery and emotion regulation strategies. Conscious Cogn 2006;15:342–50. 16230028 10.1016/j.concog.2005.09.001

[niz016-B15] Dayan P, Berridge KC. Model-based and model-free Pavlovian reward learning: revaluation, revision, and revelation. Cogn Affect Behav Neurosci 2014;14:473–92. 24647659 10.3758/s13415-014-0277-8 PMC4074442

[niz016-B16] Dehaene S, Kerszberg M, Changeux J-P. A neuronal model of a global workspace in effortful cognitive tasks. Proc Natl Acad Sci USA 1998;95:14529–34. 9826734 10.1073/pnas.95.24.14529 PMC24407

[niz016-B17] Dehaene S, Lau H, Kouider S. What is consciousness, and could machines have it? Science 2017;358:486–92. 29074769 10.1126/science.aan8871

[niz016-B18] Dehaene S, Naccache L. Towards a cognitive neuroscience of consciousness: basic evidence and a workspace framework. Cognition 2001;79:1–37. 11164022 10.1016/s0010-0277(00)00123-2

[niz016-B19] Dennett DC. Kinds of Minds: Toward an Understanding of Consciousness. New York: BasicBooks, 1996.

[niz016-B20] Dijkstra N, Bosch SE, van Gerven MA. Vividness of visual imagery depends on the neural overlap with perception in visual areas. J Neurosci 2017;37:1367–73. 28073940 10.1523/JNEUROSCI.3022-16.2016 PMC6596858

[niz016-B21] Earl B. The biological function of consciousness. Front Psychol 2014;5:697. 25140159 10.3389/fpsyg.2014.00697 PMC4122207

[niz016-B22] Fleming SM, Lau HC. How to measure metacognition. Front Hum Neurosci 2014;8. 10.3389/fnhum.2014.00443 PMC4097944 25076880

[niz016-B23] Friston K. The free-energy principle: a rough guide to the brain? Trends Cogn Sci 2009;13:293–301. 19559644 10.1016/j.tics.2009.04.005

[niz016-B24] Friston K. The free-energy principle: a unified brain theory? Nat Rev Neurosci 2010;11:127–38. 20068583 10.1038/nrn2787

[niz016-B25] Friston K, Adams R, Perrinet L, et al Perceptions as hypotheses: saccades as experiments. Front Psychol 2012;3:151. 22654776 10.3389/fpsyg.2012.00151 PMC3361132

[niz016-B26] Friston K, Kiebel S. Predictive coding under the free-energy principle. Philos Trans R Soc B 2009;364:1211–21. 10.1098/rstb.2008.0300 PMC2666703 19528002

[niz016-B27] Friston K, Rigoli F, Ognibene D, et al Active inference and epistemic value. Cogn Neurosci 2015;6:187–214. 25689102 10.1080/17588928.2015.1020053

[niz016-B28] Friston K, Samothrakis S, Montague R. Active inference and agency: optimal control without cost functions. Biol Cybern 2012;106:523–41. 22864468 10.1007/s00422-012-0512-8

[niz016-B29] Friston K, Schwartenbeck P, Fitzgerald T, et al The anatomy of choice: active inference and agency. Front Hum Neurosci 2013;7:598. 24093015 10.3389/fnhum.2013.00598 PMC3782702

[niz016-B30] Frith CD, Metzinger T. How the stab of conscience made us really conscious In: Engel AK, Friston KJ, Kragic D. (eds), The Pragmatic Turn: Toward Action-oriented Views in Cognitive Science. MIT Press, 2016, 193.

[niz016-B31] Giacino JT, Ashwal S, Childs N, et al The minimally conscious state: definition and diagnostic criteria. Neurology 2002;58:349–53. 11839831 10.1212/wnl.58.3.349

[niz016-B32] Goodale MA, Jakobson LS, Keillor JM. Differences in the visual control of pantomimed and natural grasping movements. Neuropsychologia 1994;32:1159–78. 7845558 10.1016/0028-3932(94)90100-7

[niz016-B33] Goodale MA, Milner AD, Jakobson LS, et al A neurological dissociation between perceiving objects and grasping them. Nature 1991;349:154–6. 1986306 10.1038/349154a0

[niz016-B34] Goodfellow I, Pouget-Abadie J, Mirza M, et al *Proceedings of the 27th International Conference on Neural Information Processing Systems* 2014, Vol. 2 pp. 2672–2680.

[niz016-B35] Gregory R. Brainy mind. BMJ (Clin Res Ed.) 1998;317:1693–5. 10.1136/bmj.317.7174.1693 PMC1114483 9857130

[niz016-B36] Grush R. The emulation theory of representation: motor control, imagery, and perception. Behav Brain Sci 2004;27:377–96. 15736871 10.1017/s0140525x04000093

[niz016-B37] Gulrajani I, Ahmed F, Arjovsky M, et al Improved training of Wasserstein GANs In: Advances in Neural Information Processing Systems, Long Beach, California, USA 2017, pp. 5767–5777. USA: Curran Associates Inc., 2017.

[niz016-B38] Ha D, Schmidhuber H. Recurrent world models facilitate policy evolution In: Advances in Neural Information Processing Systems 2018, pp. 2450–2462. USA: Curran Associates, Inc., 2018.

[niz016-B39] Heeger DJ. Theory of cortical function. Proc Natl Acad Sci 2017;114:1773–82. 28167793 10.1073/pnas.1619788114 PMC5338385

[niz016-B40] Hesse C, Schenk T. Delayed action does not always require the ventral stream: a study on a patient with visual form agnosia. Cortex 2014;54:77–91. 24657477 10.1016/j.cortex.2014.02.011

[niz016-B41] Hindupur A. *The GAN Zoo*, 2017 https://deephunt.in/the-gan-zoo-79597dc8c347 (14 November 2019, date last accessed).

[niz016-B42] Hohwy J. The Predictive Mind , 1st edn Oxford, UK; New York, NY, USA: Oxford University Press, 2014.

[niz016-B43] Hsieh P-J, Colas JT, Kanwisher N. Pop-out without awareness: unseen feature singletons capture attention only when top-down attention is available. Psychol Sci 2011;22:1220–6. 21852451 10.1177/0956797611419302 PMC3264049

[niz016-B44] Jeannerod M. The representing brain: neural correlates of motor intention and imagery. Behav Brain Sci 1994;17:187–202.

[niz016-B45] Kanai R, Tsuchiya N. Qualia. Curr Biol 2012;22:R392–R396. 22625852 10.1016/j.cub.2012.03.033

[niz016-B46] Kanai R, Tsuchiya N, Verstraten FAJ. The scope and limits of top-down attention in unconscious visual processing. Curr Biol 2006;16:2332–6. 17141615 10.1016/j.cub.2006.10.001

[niz016-B47] King J-R, Pescetelli N, Dehaene S. Brain mechanisms underlying the brief maintenance of seen and unseen sensory information. Neuron 2016;92:1122–34. 27930903 10.1016/j.neuron.2016.10.051

[niz016-B48] Kingma DP, Welling M. *Auto-encoding Variational Bayes* ArXiv: 1312.6114 [Cs, Stat], 2013 http://arxiv.org/abs/1312.6114.

[niz016-B49] Koch C. The Quest for Consciousness: A Neurobiological Approach , 1st edn Denver, CO: Roberts & Co, 2004.

[niz016-B50] Koch C, Massimini M, Boly M, et al Neural correlates of consciousness: progress and problems. Nat Rev Neurosci 2016;17:307–21. 27094080 10.1038/nrn.2016.22

[niz016-B51] Koch C, Tsuchiya N. Attention and consciousness: two distinct brain processes. Trends Cogn Sci 2007;11:16–22. 17129748 10.1016/j.tics.2006.10.012

[niz016-B52] Lamme VAF, Supèr H, Landman R, et al The role of primary visual cortex (V1) in visual awareness. Vision Res 2000;40:1507–21. 10788655 10.1016/s0042-6989(99)00243-6

[niz016-B53] Lau HC, Passingham RE. Unconscious activation of the cognitive control system in the human prefrontal cortex. J Neurosci 2007;27:5805–11. 17522324 10.1523/JNEUROSCI.4335-06.2007 PMC6672767

[niz016-B54] Lau H, Rosenthal D. Empirical support for higher-order theories of conscious awareness. Trends Cogn Sci 2011;15:365–73. 21737339 10.1016/j.tics.2011.05.009

[niz016-B55] Laureys S, Celesia GG, Cohadon F, et al; the European Task Force on Disorders of Consciousness. Unresponsive wakefulness syndrome: a new name for the vegetative state or apallic syndrome. BMC Med 2010;8:68. 21040571 10.1186/1741-7015-8-68 PMC2987895

[niz016-B56] Lin Z, He S. Seeing the invisible: the scope and limits of unconscious processing in binocular rivalry. Prog Neurobiol 2009;87:195–211. 18824061 10.1016/j.pneurobio.2008.09.002 PMC2689366

[niz016-B57] Manita S, Suzuki T, Homma C, et al A top-down cortical circuit for accurate sensory perception. Neuron 2015;86:1304–16. 26004915 10.1016/j.neuron.2015.05.006

[niz016-B58] Oizumi M, Albantakis L, Tononi G. From the phenomenology to the mechanisms of consciousness: Integrated Information Theory 3.0. PLoS Comput Biol 2014;10:e1003588. 24811198 10.1371/journal.pcbi.1003588 PMC4014402

[niz016-B59] Oizumi M, Tsuchiya N, Amari S. Unified framework for information integration based on information geometry. Proc Natl Acad Sci USA 2016;113:14817–22. 27930289 10.1073/pnas.1603583113 PMC5187746

[niz016-B60] Oudeyer P-Y. What is intrinsic motivation? A typology of computational approaches. Front Neurorobot 2007;1:6. 18958277 10.3389/neuro.12.006.2007 PMC2533589

[niz016-B61] Pearl J, Mackenzie D. The Book of Why: The New Science of Cause and Effect, 1st edn New York: Basic Books, 2018.

[niz016-B62] Pearson J. The human imagination: the cognitive neuroscience of visual mental imagery. Nat Rev Neurosci 2019;20:624–34. 31384033 10.1038/s41583-019-0202-9

[niz016-B63] Pennartz CMA. Consciousness, representation, action: the importance of being goal-directed. Trends Cogn Sci 2018;22:137–53. 29233478 10.1016/j.tics.2017.10.006

[niz016-B64] Piccinini G, Craver C. Integrating psychology and neuroscience: functional analyses as mechanism sketches. Synthese 2011;183:283–311.

[niz016-B65] Ramachandran VS, Hirstein W. Three laws of qualia: what neurology tells us about the biological functions of consciousness. J Conscious Stud 1997;4:429–57.

[niz016-B66] Rao RP, Ballard DH. Predictive coding in the visual cortex: a functional interpretation of some extra-classical receptive-field effects. Nat Neurosci 1999;2:79–87. 10195184 10.1038/4580

[niz016-B67] Rosenthal D. Consciousness and Mind, 1st edn Oxford, NY: Clarendon Press, 2006.

[niz016-B68] Ruffini G. An algorithmic information theory of consciousness. Neurosci Conscious 2017;1:nix019. 10.1093/nc/nix019 PMC6007168 30042851

[niz016-B69] Schmidhuber J. Curious model-building control systems. In: *[Proceedings] 1991 IEEE International Joint Conference on Neural Networks*, Vol. 2. 1991, 1458–63.

[niz016-B70] Schmidhuber J. Formal theory of creativity, fun, and intrinsic motivation (1990–2010). *[Proceedings] 1991 IEEE International Joint Conference on Neural Networks, Singapore, 1991*, pp. 1458–1463, vol. 2, doi: 10.1109/IJCNN.1991.170605.

[niz016-B71] Searle J. Biological naturalism In: Schneider S, Velmans M(eds), The Blackwell Companion to Consciousness. Hoboken, New Jersey: Wiley Blackwell, 2017, 327–36.

[niz016-B72] Seth A. A predictive processing theory of sensorimotor contingencies: explaining the puzzle of perceptual presence and its absence in synesthesia. Cogn Neurosci 2014;5:97–118. 24446823 10.1080/17588928.2013.877880 PMC4037840

[niz016-B73] Sherman M, Barrett AB, Kanai R. Inferences about consciousness using subjective reports of confidence In: Overgaard M. (ed.), Behavioral Methods in Consciousness Research. Oxford, UK: Oxford University Press, 2015, 87–106.

[niz016-B74] Soto D, Mäntylä T, Silvanto J. Working memory without consciousness. Curr Biol 2011;21:R912–R913. 22115455 10.1016/j.cub.2011.09.049

[niz016-B75] Supèr H, Spekreijse H, Lamme VA. Two distinct modes of sensory processing observed in monkey primary visual cortex (V1). Nat Neurosci 2001;4:304–10. 11224548 10.1038/85170

[niz016-B76] Sutton RS. Dyna, an integrated architecture for learning, planning, and reacting. ACM SIGART Bull 1991;2:160–3.

[niz016-B77] Tajima S, Kanai R. Integrated information and dimensionality in continuous attractor dynamics. Neurosci Conscious 2017;2017:1–9. 10.1093/nc/nix011 PMC6007138 30042844

[niz016-B78] Thagard P, Stewart TC. Two theories of consciousness: semantic pointer competition vs. information integration. Conscious Cogn 2014;30:73–90. 25160821 10.1016/j.concog.2014.07.001

[niz016-B79] Tononi G. An information integration theory of consciousness. BMC Neurosci 2004;5:42. 15522121 10.1186/1471-2202-5-42 PMC543470

[niz016-B80] Tononi G. Consciousness as integrated information: a provisional manifesto. Biol Bull 2008;215:216–42. 19098144 10.2307/25470707

[niz016-B81] Tononi G, Boly M, Massimini M, et al Integrated information theory: from consciousness to its physical substrate. Nat Rev Neurosci 2016;17:450–61. 27225071 10.1038/nrn.2016.44

[niz016-B82] Velmans M. Is human information processing conscious? Behav Brain Sci 1991;14:651–69.

[niz016-B83] Vergassola M, Villermaux E, Shraiman BI. ‘Infotaxis’ as a strategy for searching without gradients. Nature 2007;445:406–9. 17251974 10.1038/nature05464

